# Neural dynamics of inflectional and derivational processing in spoken word comprehension: laterality and automaticity

**DOI:** 10.3389/fnhum.2013.00759

**Published:** 2013-11-18

**Authors:** Caroline M. Whiting, William D. Marslen-Wilson, Yury Shtyrov

**Affiliations:** ^1^Department of Psychology, University of CambridgeCambridge, UK; ^2^MRC Cognition and Brain Sciences UnitCambridge, UK; ^3^Center of Functionally Integrative Neuroscience, Aarhus UniversityAarhus, Denmark; ^4^Centre for Languages and Literature, Lund UniversityLund, Sweden

**Keywords:** morphology, MEG, EEG, inflection, derivation, language comprehension, attention

## Abstract

Rapid and automatic processing of grammatical complexity is argued to take place during speech comprehension, engaging a left-lateralized fronto-temporal language network. Here we address how neural activity in these regions is modulated by the grammatical properties of spoken words. We used combined magneto- and electroencephalography to delineate the spatiotemporal patterns of activity that support the recognition of morphologically complex words in English with inflectional (*-s*) and derivational (*-er*) affixes (e.g., *bakes, baker*). The mismatch negativity, an index of linguistic memory traces elicited in a passive listening paradigm, was used to examine the neural dynamics elicited by morphologically complex words. Results revealed an initial peak 130–180 ms after the deviation point with a major source in left superior temporal cortex. The localization of this early activation showed a sensitivity to two grammatical properties of the stimuli: (1) the presence of morphological complexity, with affixed words showing increased left-laterality compared to non-affixed words; and (2) the grammatical category, with affixed verbs showing greater left-lateralization in inferior frontal gyrus compared to affixed nouns (*bakes *vs. *beaks*). This automatic brain response was additionally sensitive to semantic coherence (the meaning of the stem vs. the meaning of the whole form) in left middle temporal cortex. These results demonstrate that the spatiotemporal pattern of neural activity in spoken word processing is modulated by the presence of morphological structure, predominantly engaging the left-hemisphere’s fronto-temporal language network, and does not require focused attention on the linguistic input.

## INTRODUCTION

Successful speech comprehension involves extracting linguistic information from a spoken input and accessing a unique representation from the mental lexicon. In mapping from speech to meaning, converging evidence from behavioral, neuroimaging, and neuropsychological studies indicates that the grammatical structure of a word is automatically detected and segmented – e.g., *darkness* is broken down**into two morphemes, the stem *dark* and the affix -*ness* (Taft and Forster, 1975; Marslen-Wilson et al., 1994 see Rastle and Davis, 2008 for review). This has motivated longstanding questions about how lexical representations are organized and accessed, in particular for words containing more than one morpheme^1^. Morphological complexity plays a key role in languages such as English by introducing systematic and productive elements to the language, broadening the range of possible meanings through the use of multiple morphemes within a word. A critical question in this study will be how the language system identifies and processes this linguistic complexity as the speech signal unfolds.

We examine two types of affixes in English, inflectional (*-s*) and derivational (*-er*), both of which combine with a stem to form a morphologically complex word.^2^ Forms containing an inflectional suffix are semantically transparent, such that the meaning of the complex form is predictable from the meaning of the stem (e.g., *jump-jumps-jumped*). It has been argued that inflections create a new form but not a new lexical entry (Clahsen et al., 2003). Derivational affixes function in changing the meaning and in many cases the grammatical category of the stem (e.g., *farm-farmer*). To date, extensive evidence from masked priming in the visual domain supports the claim for automatic morphological segmentation (Rastle et al., 2000,^2004^; Longtin et al., 2003; Longtin and Meunier, 2005; Marslen-Wilson et al., 2008), where* any* word containing a potential stem and affix is segmented. This work has primarily focused on derived forms, but research on inflected forms – often centered on distinctions between regular and irregular past-tense processing – has also pointed to early morphological decomposition (Meunier and Marslen-Wilson, 2004; Crepaldi et al., 2010). Converging evidence for processing of inflected forms has come from spoken word comprehension. Spoken forms ending in the characteristic pattern of regular inflection in English – a final coronal consonant (d, t, s, z) that agrees in voicing with the preceding phoneme (e.g., *played *and *plays* as opposed to vowel–consonant voicing mismatch in *plate *and *place*) – will trigger automatic morphological decomposition (Tyler et al., 2005; Post et al., 2008). Though this appears counterproductive for words such as *corner* or *trade*, where a decompositional reading of *corn* + -*er* or *tray *+ -*ed *has no relationship to the meaning of the whole form, it suggests a tuned sensitivity of the language system to morphological structure.

To address the neural foundations of this automatic morphological process, it is essential to use a brain imaging technique which can provide not only spatial but also temporal precision. For this reason, we use concurrent magnetoencephalography (MEG) and electroencephalography (EEG) recordings of brain responses to morphologically simple and complex words. In the visual domain, converging cross-linguistic evidence using EEG has pointed to specific processes linked to the presence of morphological complexity in the time window of the N400 (Münte et al., 1999; Rodriguez-Fornells et al., 2001; Lavric et al., 2007; Lehtonen et al., 2007), with additional studies showing earlier effects between 150 and 300 ms (Morris et al., 2008; Lehtonen et al., 2011; Lavric et al., 2012; Morris and Stockall, 2012). Recent MEG evidence has revealed early effects before 200 ms (Zweig and Pylkkänen, 2009; Lewis et al., 2011), as well as effects peaking at 400 ms (Vartiainen et al., 2009). Taken as a whole, these studies provide evidence for sensitivity to potential morphological structure, with the work on derived forms showing that complex and pseudo-complex forms like *farmer* and *corner* produce a similar neural pattern relative to orthographic controls such as *scandal *(*scan* + non-affix *–dal*; Morris et al., 2008; Lehtonen et al., 2011; Lavric et al., 2012). These findings have been taken as evidence for automatic morphological segmentation independent of word meaning, confirming the behavioral masked priming effects.

Evidence for blind morphological decomposition does not, however, require a decompositional representation for all words containing morphological structure – and indeed would not be appropriate for pseudo-affixed words such as *corner*. Dual-route accounts have been proposed which argue for decompositional processes, but allow for the co-existence of whole-word and morphologically decomposed representations (Caramazza et al., 1985; Marslen-Wilson et al., 1994; Schreuder and Baayen, 1995). This presupposes a level of processing where forms are accessed in terms of their constituent morphemes, but does not assume all complex words are accessed through parsing. Electrophysiological evidence for dual-route recognition has been demonstrated through sensitivity to surface frequency and the relationship between stem and suffix (transition probability), suggesting that both whole form and morphological factors modulate early stages of word processing (Lewis et al., 2011). Features of the affix are thought to play a key role in determining whether a form is represented decompositionally or as a full form, including word formation type (inflected vs. derived) and the productivity of the affix (Bertram et al., 2000).

There is accumulating neuroimaging and neuropsychological evidence to suggest that the presence of an inflectional ending engages left hemisphere fronto-temporal regions, with specific involvement of the left inferior frontal gyrus (IFG; Laine et al., 1999; Longworth et al., 2005; Tyler et al., 2005; Lehtonen et al., 2006; Bozic et al., 2010). Derivationally complex forms appear to show a distinct neural pattern, engaging a more bilateral system (Meinzer et al., 2009; Leminen et al., 2011; Bozic et al., 2013), and suggesting that lexical access to derivations may be achieved via the full forms. We aim to detail these putative differences in brain activation dynamics by comparing EEG/MEG activation elicited by inflections and derivations in a tightly controlled stimulus set. We focus in this study on the initial stages of morphological processing involved in identifying complexity. If there is rapid morphological segmentation, as has been argued in the visual domain (see Rastle and Davis, 2008 for review), we would hypothesize that this process will be triggered for both types of affixes (inflectional and derivational) once phonological cues to the presence of the affix are identified.

Particular challenges arise when addressing morphological processing in the auditory domain. Unlike written text, where there are discrete letters available simultaneously to the reader, spoken language is uttered in a continuous stream. The listener must recognize linguistic units within a stream that is evolving over time, with new information constantly arriving to the auditory system. Models of spoken word processing state that listeners are able to recognize words before they have finished hearing them (Marslen-Wilson and Welsh, 1978; Grosjean, 1980), where multiple candidates compete for selection until the speech input is uniquely identifiable. The notion of simultaneous activation of all potential candidate words is a fundamental concept in many spoken word recognition models (Marslen-Wilson and Welsh, 1978; McClelland and Elman, 1986; Norris, 1994). Thus, an important issue is determining the point in the speech signal at which there is sufficient information to determine its correct identity, in particular when considering the relationship between the meaning of the stem and the meaning of the complex form (*jump-jumps, farm-farmer, corn-corner*). By tracking the time course of spoken word comprehension using time-resolved MEG/EEG, it is possible to time-lock neural responses precisely to the suffix onset and thus investigate how the suffix triggers segmentation once it can be identified in the speech signal.

In delineating the neural systems underlying speech comprehension using fMRI, a bilateral fronto-temporal network has been shown to be engaged in the processing of spoken words, including superior and middle temporal regions which are linked to the processing of lexical meaning (Binder et al., 2000; Scott et al., 2000; Davis and Johnsrude, 2003; Hickok and Poeppel, 2007). A further left-lateralized subsystem of this network has been implicated in the processing of morphological complexity, comprising left-hemisphere frontal, temporal, and parietal regions (Friederici et al., 2003; Marslen-Wilson and Tyler, 2007; Bozic et al., 2010). Thus, by manipulating the presence or absence of potential morphological complexity, we can investigate how these bilateral and left-lateralized networks are activated during spoken word comprehension. Once evidence has accumulated that a potential affix is present in the speech signal, we would predict that processing should automatically shift to the left-lateralized fronto-temporal system.

To address these issues neurophysiologically, it is necessary to use brain responses that reflect automatic processing, provide accurate information on the time course of stimulus-specific processing in the brain, and that are sensitive to the linguistic properties of the stimuli. For these reasons, the present study involves the use of the mismatch negativity (MMN), a neural response component elicited by rare unexpected changes in the auditory stream. The paradigm consists of an oddball design in which a sequence of a frequent “standard” stimulus is occasionally replaced by a rare “deviant” stimulus (Näätänen et al., 1978). It has been argued that the MMN – typically a negative deflection peaking 100–200 ms after the onset of the change between deviant and standard – can reflect the activation of experience-dependent auditory memory traces (Näätänen et al., 1997).

Crucially for our study, the mismatch response is sensitive to linguistic sounds such as syllables and words, resulting in an increased left-lateralized response for language deviants (Näätänen et al., 1997; Shtyrov et al., 2005). The amplitude of the MMN shows a specific increase for real words compared to acoustically matched pseudowords (Korpilahti et al., 2001; Pulvermüller et al., 2001). This lexical enhancement effect is explained by the existence of cortical memory traces that are automatically activated for known words in passive oddball sequences, but fail to activate for pseudowords that are not stored in the lexicon (Pulvermüller et al., 2001; Näätänen et al., 2007). Importantly, the timing of the mismatch response has been linked to behaviourally determined word-specific recognition times (Pulvermüller et al., 2006) whilst temporal patterns of local cortical activation spread show fine-tuned specificity for linguistic stimulus properties (Pulvermüller and Shtyrov, 2009).

Evidence from English inflectional morphology has shown that the mismatch response is modulated by grammatical changes due to the presence of morphological structure, with effects emerging in left-lateralized perisylvian areas for affixed deviants as compared to stems (Shtyrov and Pulvermüller, 2002); similar activity patterns were found for MMN responses elicited by differences in morphological structure in Arabic (Boudelaa et al., 2010). Our focus in this study is on the initiation of morphological segmentation of potentially complex forms, which is argued to be triggered automatically (e.g., Tyler et al., 2002; Post et al., 2008). A key advantage of the MMN paradigm is the ability to record neural responses elicited in the absence of focused attention on the auditory stream, enabling an investigation into early stages of spoken word recognition and the initiation of morphological processing before strategic effects or conscious processing of the word forms have taken place.

The MMN paradigm relies on a small set of items, implying that caution is needed in generalizing MMN results to the entire language. However, it offers a number of benefits, which make it an attractive tool for studies of spoken word recognition. It allows for ruling out acoustic confounds by incorporating the same acoustic/phonological contrast (e.g., addition of the same consonant) into different linguistic contexts which can themselves be tightly matched acoustically. By determining the time-point of standard-deviant divergence (such as addition of an affix here), neural responses can be aligned precisely allowing for a direct comparison between different morphological conditions. Finally, as mentioned above, it is an automatic response in that its elicitation does not depend on focusing attention on stimuli or engaging in a stimulus-related task.

In the present study, we include a matched set of inflected, derived and non-affixed forms. The inflectionally complex forms (*bakes, beaks*) allow us to examine how neural activity in the language system is modulated by the presence of an affix which results in a fully transparent form. Inflectional suffixes do not modify the meaning of the stem, and it has been argued that regularly inflected forms are represented and accessed compositionally (Pinker and Ullman, 2002; Marslen-Wilson and Tyler, 2007). We predict that inflected forms should trigger automatic decomposition, engaging a left-lateralized network including inferior frontal cortex compared to non-affixed forms (Tyler et al., 2005). Converging MMN findings show a left-lateralized response to inflected forms at ~150 ms (Shtyrov and Pulvermüller, 2002; Shtyrov et al., 2005), indicating that the mismatch response can reveal specific memory trace activations in the neural subsystems involved in morphological decomposition.

Further, such a stimulus design allows us to directly contrast the verb (*bakes*) and the noun (*beaks*) inflection in order to examine potential differences related to grammatical class (signaling agreement as opposed to nominal plural).**Differential noun vs. verb processing has been suggested in previous studies, where inflected verbs have revealed an increased left-lateralized distribution compared to inflected nouns, with key involvement of left inferior frontal cortex (Shapiro and Caramazza, 2003; Tyler et al., 2004; Longe et al., 2007). Though both forms are morphologically complex and would require segmentation into stem and suffix, it has been argued that verbs and nouns differentially engage the neural systems involved in morphological processing when they are inflected. This has been linked to differences in grammatical function of verbs and nouns in English, where verbs can be associated with a greater range of inflections to mark number, tense and person (unlike nouns, which only mark number), thus playing a greater role in the structural interpretation of a sentence (Tyler et al., 2004). However, the automaticity of this neural distinction between word classes remains unexplored. In the present study, we hypothesize increased engagement of left fronto-temporal regions for suffixed verbs compared to nouns, in particular in left inferior frontal cortex.

Using the derivational suffix *-er*, we investigate a further contrast between semantically transparent and pseudo-affixed word forms (*baker* vs. *beaker*) in order to examine whether morphological processing is indeed unaffected by the lexical appropriateness of the segmentation, as indicated by the previous behavioral investigations. Like the inflected forms, we would predict automatic segmentation of complex and pseudo-complex forms, with both derived forms patterning with the inflected forms compared to non-affixed forms. This would indicate the existence of discrete neural networks for automatic morphological processing which should be engaged for all forms containing potential complexity (e.g., Morris et al., 2008; Lehtonen et al., 2011; Lavric et al., 2012).

The two affixed conditions (*bakes/baker, beaks/beaker*) are contrasted with non-affixed forms (*bacon/beacon*) that embed the same (false) stems but should not trigger any attempts at segmentation as no affix is present. These non-complex forms are likely to engage a more bilateral cortical distribution, since morphological processes may not be engaged when no clues for morphological segmentation (such as a valid suffix) are present (Bozic et al., 2010). In addition, we include a control condition aimed at assessing acoustic/phonological effects by incorporating the same deviant contrasts in a meaningless pseudoword (*boke*). This provides a way of assessing whether effects are due to processing of low-level acoustic changes, rather than morphological processing.

In summary, the aim of this work is to examine how the spatiotemporal dynamics of word processing are modulated once a potential affix is identified in the speech signal. We focus on pinpointing when and where morphological information is accessed, and whether this is done automatically in the absence of attention, using the fine-grained spatiotemporal resolution of combined magneto- and electroencephalography (MEG–EEG). We address two issues of morphological processing: contrasting suffixed and non-suffixed forms, as well as inflected and derived forms, the latter comprising both semantically transparent and opaque derivations. We predict increased left fronto-temporal engagement for all morphologically complex forms compared to simple forms, regardless of word meaning, triggered by the presence of an inflectional or derivational suffix. Furthermore, with the inflected forms we examine processing of grammatical category, contrasting noun and verb forms. Verbal *-s* forms should elicit more left fronto-temporal activation, in particular in IFG, compared to nominal -*s* forms. To assess this potential shift in left hemisphere engagement for morphologically complex forms, we incorporate a laterality analysis (Shtyrov et al., 2005) to examine hemispheric differences across the complex and non-complex forms. The MMN paradigm has revealed increased left-lateralization for language stimuli (Näätänen et al., 1997), and we would predict this laterality should increase for morphologically complex forms compared to simple forms, and for verbs compared to nouns, both properties which have been shown to modulate the degree of left fronto-temporal activity. In this way we can examine how the addition of a suffix modulates the spatiotemporal pattern of word recognition as the speech signal unfolds, as well as the networks that support recognition of morphologically simple and complex spoken words.

## MATERIALS AND METHODS

### SUBJECTS

Fifteen subjects (13 female) took part in the experiment. All were right-handed (handedness tested according to Oldfield, 1971; range: 85–100%) native British English speakers between the ages of 19–34 (mean age of 24.9) with normal hearing, normal or corrected-to-normal vision, and no history of neurological disease, who gave written consent to take part and were paid for their time.

### MATERIALS

Standards and deviants were selected on the basis that acoustic differences would be minimized while manipulating lexicality, semantic transparency (relationship between stem and whole form), and morphological complexity (the presence of a potential suffix). Two word conditions (*bake, beak*) and one pseudoword condition (*boke*) were presented as standards in separate experimental blocks. Three deviants were created by adding an inflectional affix (*-s*), a derivational affix (*-er*)*, *and a non-affix (*-on*) to each of the standards (see **Table 1**). Crucially, the addition of *-er* produced a semantically transparent or opaque meaning in relation to the stem (*baker *vs. *beaker*). Both inflected forms (*bakes, beaks*) produced a valid complex form but differed in word class (verb vs. noun). Stimuli were matched on spoken wordform frequency, taken from the Celex database (Baayen et al., 1995), and neighborhood size (*N*).

**Table 1:** Standards and deviants used in MMN study.

Standard	*bake*	*beak*	*boke
deviant 1 (*-er*)	*bakes*	*beaks*	*bokes
deviant 2 (*-s*)	*baker*	*beaker*	*boker
deviant 3 (*-on*)	*bacon*	*beacon*	*bokon

* indicates pseudoword.

Unaffixed stem stimuli *(bake, beak, boke)* were spoken by a female native British English speaker. Multiple versions of the standards were recorded, and the selected stimuli were closely matched on pitch/fundamental frequency, intensity and duration. The [b + vowel] segment was cut from each standard and served as the base form for all stimuli in the experiment. These base forms were adjusted to be of equal length (165 ms); they were also normalized for their peak amplitude. Endings for the standards and deviants were taken from recordings of the words *wreck*, *wrecks*, *wrecker,* and *reckon*; thus, the speaker produced the endings in the context of real words without a specific co-articulation bias toward any vowel used in the test stimuli. Multiple tokens of these words were also recorded and the selected words were closely matched on the pitch, intensity and duration to the main test stimuli. Each [k + ending] was spliced after the [b + vowel] following a 75 ms pause, which signaled the closure period before the release of the [k] typical of stop-consonants in the English language. The duration of the deviant endings were also adjusted to be of equal length starting from the [k] release.

**FIGURE 1:**
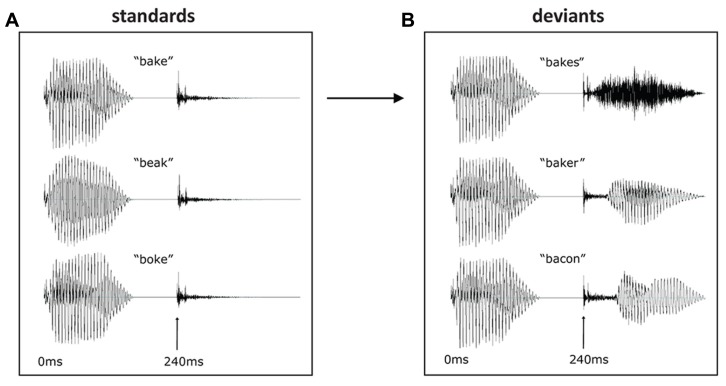
**Waveforms of stimuli used as standards (A)** and deviants **(B)**. Deviants displayed are for the *bake* condition, with the three deviant endings following the same stem. The *beak* and *boke* conditions were constructed using the same endings. At 240 ms the [k] is released in each condition, marking the deviation point from the standard.

Within each condition, the same [b + vowel] was used, and within each deviant set, the same [k + ending] was used. Thus, the stimuli of a given condition (i.e., all *bake* forms) were identical until the release of the [k]. This occurred at 240 ms post-stimulus onset, and all deviants were 460 ms long in total (see **Figure 1**). In this way, a set of naturally sounding but strictly controlled stimuli were obtained that were matched for acoustic–phonetic properties between conditions; furthermore, the deviant-standard contrasts (the critical feature determining purely acoustic MMN) were identical across the three main sets. At the same time, the context in which these contrasts occurred was systematically modulated, allowing us to rule out any acoustic confounds and concentrate on the linguistic context effects.

### PROCEDURE

Stimuli were presented pseudo-randomly in blocks of approximately 20 min in length, with short pauses between blocks and in the middle of each block. The order of the conditions was randomized across subjects. The pseudo-randomization within each block was done to ensure that at least two standards appeared between every deviant, and the order of the deviants within the blocks was completely random. Each stimulus was presented for 460 ms with a jittered inter-trial offset-to-onset interval of 460–500 ms. For each condition, 100 trials of each of the three deviants were presented in the context of 900 standards, constituting 25% deviants (8.3% of each) and 75% standards. Ten filler trials of the standard stimulus were used at the beginning of each block to build up a representation of the standard, and were not included in the average event-related field. Every standard that appeared after a deviant was also discarded, as it might produce a change detection response of its own when immediately following the deviant.

Stimuli were presented binaurally through non-magnetic earpieces attached to plastic tubes while subjects were seated in front of a screen inside a dimly lit, magnetically shielded room. Before the experiment began, subjects were given a hearing test to ensure they could hear sounds equally well in each ear. Subjects were instructed to attend to a silent video during the experiment and did not perform a task on the stimuli, which they were instructed to ignore. They were told there would be a questionnaire following the experiment on details concerning the film, and all subjects scored at least 90% on the questionnaire. The experiment was run using E-Prime 1.0 (Psychology Software Tools, Sharpsburg, PA, USA) and lasted approximately 60 min.

### DATA ACQUISITION

Concurrent MEG–EEG data were acquired at a sampling rate of 1000 Hz (passband 0.10–330 Hz), with triggers placed at the onset of each stimulus. Neuromagnetic signals were recorded continuously with a 306-channel (102 magnetometers and 204 planar gradiometers) Vectorview MEG system (Elekta Neuromag, Helsinki, Finland). Electrical activity was recorded using a 70-channel EEG cap (Easycap, Herrsching, Germany), using a reference electrode on the nose. Prior to recording, five electromagnetic coils were positioned on the head and digitized along with the EEG electrodes using the Polhemus Isotrak digital tracker system (Polhemus, Colchester, VT, USA) with respect to three standard anatomical landmarks (nasion, left and right pre-auricular points). During the recording, the position of the magnetic coils was continuously tracked using continuous head position identification (cHPI), providing information on the exact head position within the MEG dewar for later movement correction. Four electrooculogram (EOG) electrodes were placed laterally to each eye and above and below the left eye to monitor horizontal and vertical eye movements during the recording.

### PRE-PROCESSING

Continuous raw data were pre-processed off-line with MaxFilter (Elekta Neuromag) implementation of signal-space separation (SSS) technique with a temporal extension (tSSS; Taulu and Simola, 2006), which minimizes movement artifacts and effects of magnetic sources outside the head. Averaging was performed using the MNE Suite (Athinoula A. Martinos Center for Biomedical Imaging, Boston, MA, USA). Epochs containing gradiometer, magnetometer, or EEG/EOG peak-to-peak amplitudes larger than 3000 fT/cm, 6500 fT, or 200 μV, respectively, were rejected. Trials were averaged by condition with epochs generated from -50 to 500 ms from the [k] release (at 240 ms after stimulus onset), at which point the standard and deviant stimuli started to diverge. Averaged data were low-pass filtered at 45 Hz and baseline corrected using the -50 to 0 ms interval before the divergence point. This interval was selected as it falls within the closure period preceding the [k] release (a silent period of 75 ms), and the standard and all the deviants are identical up to this point, thus there should not be any differences before this time point except random noise-related variations that should be removed using the baseline-correction procedure. The average response for the standards was subtracted from the three associated deviants to produce the MMN. For sensor-level analysis, tSSS was used to transform MEG data to the head position coordinates of the subject with the median head position within the helmet, to minimize transformation distance.

### SENSOR-LEVEL ANALYSIS

Analyses at the sensor level were conducted on EEG, gradiometers, and magnetometers separately using the sensor-space statistical parametric mapping (SPM) SensorSPM analysis method implemented in SPM5 (). EEG and magnetometer data were used as such, whilst for each pair of gradiometer channels, a vector sum was calculated reconstructing the field gradient from its two orthogonal components and its amplitude (computed as a square root of the sum of squared amplitudes in the two channels) was used in further analysis. For each subject and condition, a series of *F*-tests were performed on a three-dimensional topography (2D sensors by 1D time) image. Each contrast results in a SPM, in which clusters of contiguous suprathreshold voxels are corrected using Random Field Theory (Kiebel and Friston, 2004). The 3D images were thresholded at a voxel level of *p* < 0.005, and corrected for cluster size at *p* < 0.05. These clusters could extend in space (distributed across the topography) and in time. This made it possible to compare conditions across every sensor over time while still correcting for multiple comparisons, allowing us to investigate a wider spatiotemporal array (Shtyrov et al., 2012). This provides a conservative approach to defining significant effects, avoiding any bias inherent to conventional visual inspection.

### MRI ACQUISITION AND SOURCE ESTIMATION

MPRAGE T1-weighted structural images with a 1 mm × 1 mm × 1 mm voxel size were acquired on a 3-Tesla Trio Siemens Scanner for each subject (repetition time [TR] = 2250 ms, echo delay time [TE] = 2.99 ms, flip angle 9, field of view [FOV] = 256 mm × 240 mm × 192 mm), which were used for source reconstruction of the cortical surface using FreeSurfer (Athinoula A. Martinos Center for Biomedical Imaging). The L2 minimum-norm estimation (Hämäläinen and Ilmoniemi, 1994) technique was applied for source reconstruction as implemented in the MNE Suite. A three-layer boundary element model (scalp, inner skull, outer skull) was created for each subject and was used to compute the combined MEG + EEG forward solutions. An average cortical solution was created from the fifteen subjects, and data from individual subjects were morphed to this cortical surface in 5 ms time-steps. The cortical representation provided by FreeSurfer was decimated to 10,242 dipoles per hemisphere, providing, at every time-step, source estimates for over 20,000 dipoles.

Regions of interest (ROIs) were anatomically defined based on the Desikan–Killiany atlas of the brain (Desikan et al., 2006) as implemented in the FreeSurfer package, with the exception of the temporal regions which were subdivided into an anterior and posterior region (pre-defined ROIs extend the entire length of the temporal lobe). ROIs were defined on the average cortical surface, and for each subject the mean value for all dipoles from within each region was extracted for statistical analysis. Selected ROIs were: superior and middle temporal gyrus (STG and MTG, respectively) and IFG. Time windows were defined by the results from the 3D SensorSPM analyses where significant effects were found, and were subject to further statistical analysis using repeated measures ANOVAs with condition and ROI as within-subject factors. Source-level results are visualized on the inflated cortical surface of the average subject’s brain.

### LATERALITY ANALYSIS

Lateralization at the source level was calculated using a laterality coefficient *Q* as previously applied in psychoacoustic research and in MEG (e.g., Shtyrov et al., 2000,^2005^; Holland et al., 2012):

$$Q=\frac{\left(A_{1}-A_{r}\right)}{{(A}_{1}-A_{r})}\times 100\%,$$

where *A*_l_**and* A*_r_ are the mean amplitude across vertices in the left and right hemispheres, respectively. In this way we could assess the degree of lateralization for each condition and compare across deviant types by removing any differences in absolute magnitude. Statistical analysis was carried out using repeated measures ANOVAs, with condition and ROI as within-subject factors.

## RESULTS

**FIGURE 2:**
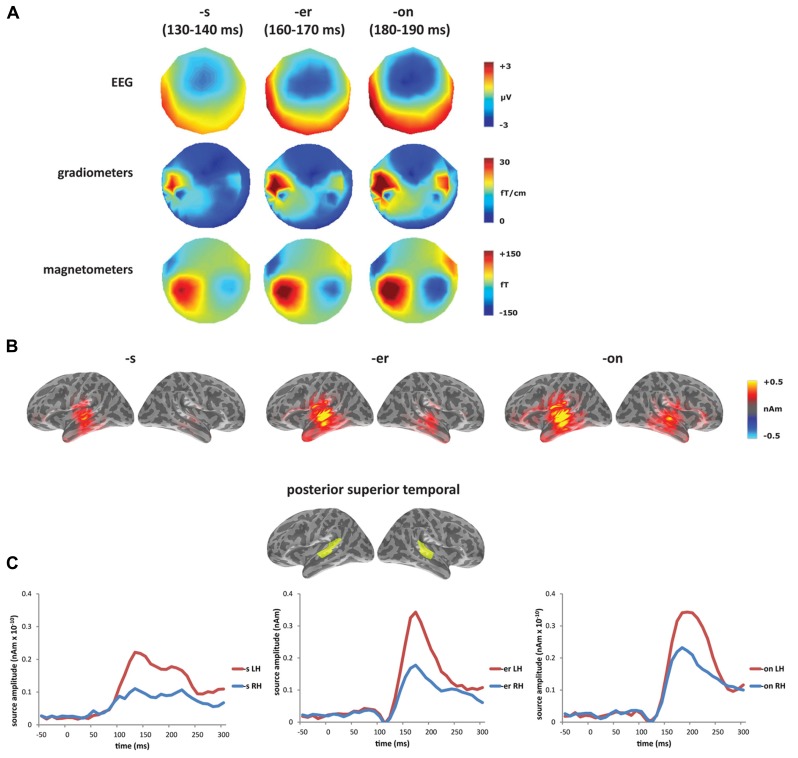
**Mismatch negativity response averaged across word conditions (*bakes*/*beaks*, *baker*/*beaker*, *bacon*/*beacon*):** **(A)** sensor level (EEG, gradiometers and magnetometers) and **(B)** source level (L2 minimum norm estimate using combined MEG + EEG) for [-s] deviant (130–140 ms), [-er] deviant (160–170 ms), and [-on] deviant (180–190 ms). **(C)** Time course of source-level activity in left and right posterior superior temporal gyrus for three deviant types.

In the presentation of the results, sensor-level results are presented separately for gradiometers, magnetometers, and EEG. **Figure 2** shows the MMN responses averaged across word conditions (*bake* and *beak*) at the sensor and source level, with the MMN defined as the peak between 100 and 200 ms with a major source in posterior temporal cortex. The zero time point was placed at the release of the [k], which was equivalent across conditions. The [-s] deviant had the earliest mismatch response, peaking at approximately 135 ms, while the [-er] deviant peaked at 165 ms and the [-on] deviant at 185 ms. As expected for word deviants, all three conditions showed a left-lateralized MMN, with largest activation within left temporal sensors in MEG and fronto-central electrodes in EEG. The combined MEG–EEG source solutions, seen in **Figure 2B**, confirmed this left-lateralized response, which localized primarily to posterior superior temporal cortex (**Figure 2C**).

**FIGURE 3:**
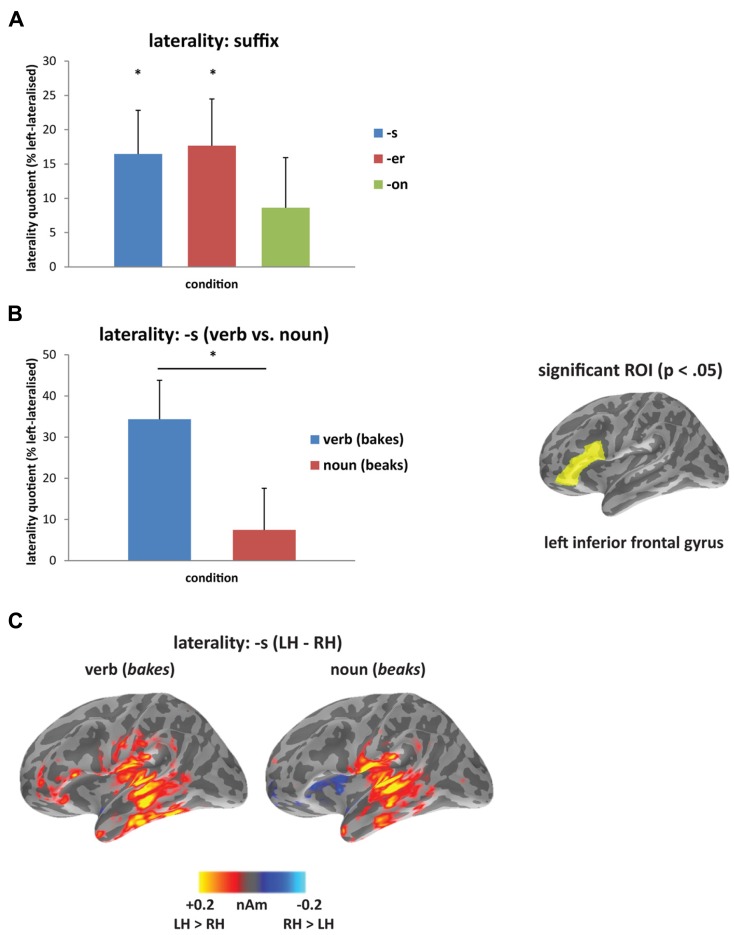
**Laterality analysis**: **(A)** contrasting affixed ([-s], [-er]) and non-affixed ([-on]) deviants, showing increased left-lateralization for affixed deviants (asterisk signifies laterality significantly greater than zero at *p*<0.05); and **(B)** contrasting verb (*bakes*) and noun (*beaks*) deviants, showing increased left-lateralization for the verb compared to the noun from 160 to 240 ms (asterisk signifies *p*<0.05), and at right, the region-of-interest (inferior frontal gyrus) showing significant lateralization. **(C)** Source activation for [-s] deviants from 160 to 240 ms, displaying the difference in amplitude between left and right hemispheres (LH minus RH at each vertex; yellow/red indicates greater left hemisphere activity; blue indicates greater right hemisphere activity).

In the laterality analysis, a 30-ms window around the peak of each mismatch response was used in order to compare across deviant conditions with differing onset latencies. We included frontal and temporal regions bilaterally, which covers the main sources of the mismatch response across the three deviant types (see **Figure 2B**), and which encompasses ROIs that have previously been implicated in morphological processing (Tyler et al., 2005; Lehtonen et al., 2006; Marslen-Wilson and Tyler, 2007; Bozic et al., 2010). Comparing the three deviants averaged across the two stems (*bakes/beaks*, *baker/beaker*, and* bacon/beacon*), there was a significant main effect of condition (*F*_(1,14)_ = 5.62, *p* < 0.05), but no effect of ROI (*F*_(4,56)_ = 1.60, *p* > 0.05) and no interaction between the two factors (*F* < 1). The effect of condition showed increased left-lateralization for the [-s] and [-er] deviants compared to [-on] (*p* < 0.05), as seen in **Figure 3A**. Based on the lack of a main effect of ROIs, we collapsed data across the five ROIs, which showed that the left-lateralization for the [-er] and [-s] conditions was in itself significant (i.e., greater than zero; (*t*_(14)_ = 2.58, *p* < 0.01 and *t*_(14)_ = 2.59, *p* < 0.05, respectively), and was not significant for the [-on] condition (*t*_(14)_ = 1.18, *p* > 0.05; two-tailed).

Within individual affix types (*bakes *vs. *beaks*, *baker *vs. *beaker, bacon *vs. *beacon*), the inflected [-s] forms were the only words to reveal a difference in laterality, with the verbal form *bakes* showing a more left-lateralized response compared to the nominal form *beaks* (**Figures 3B, C**). There was no significant main effect of condition (*F *< 1) or of ROI (*F*_(4,56)_ = 1.21, *p* > 0.05), but there was a significant interaction between condition and ROI (*F*_(__4__,__56__)_ = 2.96, *p* < 0.05) from 160 to 240 ms. We assessed this interaction statistically by carrying out a series of planned comparisons, showing greater laterality in IFG for the verb compared to the noun (*F*_(1,14)_ = 5.30, *p* < 0.05). The timing of this effect corresponds to the second half of the mismatch response for the [-s] deviants (see **Figure 2B**). **Figure 3C** demonstrates the difference in amplitude between the two hemispheres from 160 to 240 ms (LH minus RH at each vertex), with yellow/red indicating increased left hemisphere activity, and blue indicating increased right hemisphere activity. As revealed by the laterality analysis, the verb deviant showed increased left hemisphere activity in frontal and temporal areas.

### WORD–PSEUDOWORD

**FIGURE 4:**
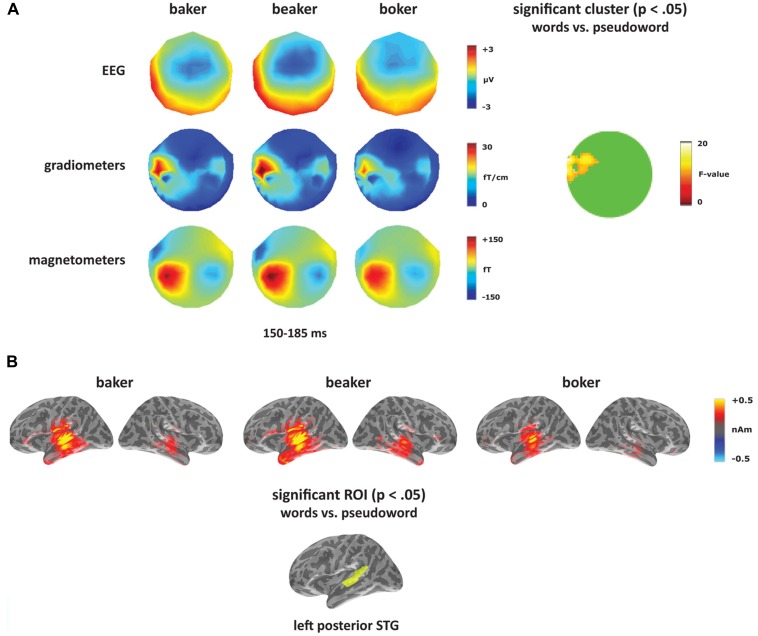
**Word–pseudoword contrast: [-er].** **(A)** Topographies for [-er] deviants (*baker, beaker, boker*) from 150 to 185 ms, and the location of the significant cluster from the sensor analysis. **(B)** Source activation for [-er] deviants from 150 to 185 ms, and the significant ROI from the source analysis (left posterior STG).

To test for a lexical enhancement effect (e.g., Pulvermüller et al., 2001), each deviant type ([-er], [-s], and [-on]) was analyzed separately contrasting the two word conditions (*bake, beak*)**with the pseudoword (*boke*). The [-er] deviants elicited a significant effect in the gradiometers within left temporal sensors with a greater response to the two word conditions compared to the pseudoword condition (see **Figure 4A**). The cluster was significant from 150 to 185 ms with a peak at 165 ms, which corresponds to the timing and the topography of the mismatch response in the [-er] deviants. Though this predominantly gradiometer-driven effect did not reach significance in the magnetometers or EEG, the topographies in **Figure 4A** showed a greater response to the word conditions (more negative for EEG) compared to the pseudoword condition across the time window of the mismatch response. No other time windows were significant. Source analyses were performed on time windows from the sensor analysis where significant effects were found. Using combined MEG and EEG at the source level, the [-er] word–pseudoword contrast (*baker, beaker *vs. *boker*) localized primarily to left posterior temporal cortex (**Figure 4B**). Significant effects of condition (*F*_(1,14)_ = 5.30, *p* < 0.05), ROI (*F*_(4,56)_ = 12.61, *p* < 0.001) and the interaction of condition and ROI (*F*_(4,56)_ = 2.89, *p* < 0.05) emerged in the left hemisphere from 150 to 185 ms. Planned comparisons showed increased amplitude for words compared to pseudowords in left posterior STG (*F*_(1,14)_ = 11.35,* p* < 0.005). In the right hemisphere, there was a significant effect of ROI (*F*_(4,56)_ = 3.73, *p* < 0.01), but no significant effect of condition (*F*_(1,14)_ = 2.53, *p* > 0.05) and no interaction between the two factors (*F *< 1).

**FIGURE 5:**
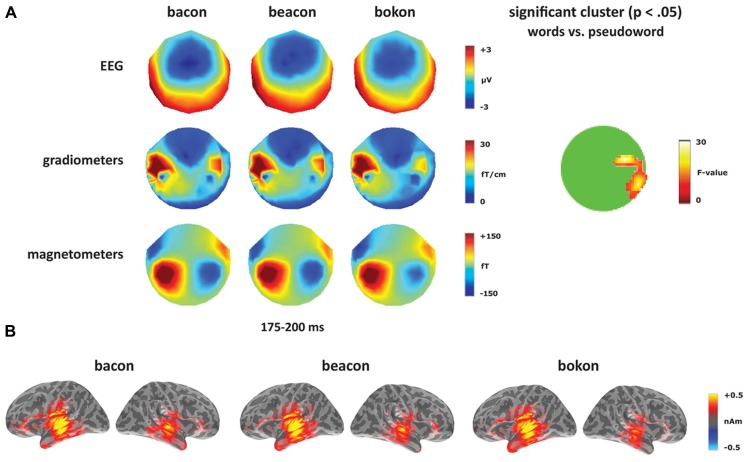
**Word–pseudoword contrast: [-on].** **(A)** Topographies for [-on] deviants (*bacon, beacon, bokon*) from 175 to 200 ms, and the location of the significant cluster from the sensor analysis. **(B)** Source activation for [-on] deviants from 175 to 200 ms.

Turning to the unaffixed [-on] deviants (**Figure 5**), these revealed a significant cluster from 175 to 200 ms within anterior right temporal gradiometers, corresponding to the timing of the [-on] mismatch response (see **Figure 5A**). Unlike the sensor-level analysis, no source ROIs showed a significant lexicality effect for the [-on] word–pseudoword contrast (*bacon, beacon *vs. *bokon*). In the left hemisphere, there was a significant effect of ROI (*F*_(4,56)_ = 12.14, *p* < 0.001) but no effect of condition (*F* < 1) or an interaction between condition and ROI (*F *< 1). In the right hemisphere, there was an effect of ROI (*F*_(4,56)_ = 5.39, *p* < 0.001), but no effect of condition (*F*_(1,14)_ = 1.38, *p* > 0.05) or an interaction between the two factors (*F* < 1).

### DERIVATIONAL TRANSPARENCY CONTRAST: BAKER vs. BEAKER

**FIGURE 6:**
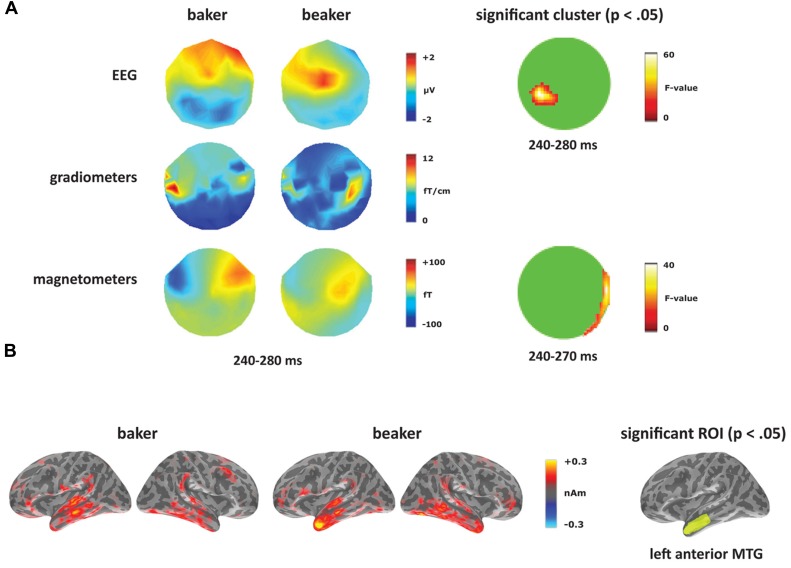
**Derivational transparency contrast: *baker* vs. *beaker***. **(A)** Topographies from 240 to 280 ms and significant clusters from sensor-level analyses. **(B)** Source activation for [-er] word deviants from 240 to 280 ms, and the significant ROI from the source analysis (left anterior MTG).

At the sensor level, the two word conditions were contrasted for each deviant type separately. Within the [-er] deviants (corresponding to the derivational affix), the words elicited a significant difference starting at 240 ms (see **Figure 6A**). In the magnetometers, the semantically opaque deviant (*beaker*) showed increased activity within right-hemisphere sensors compared to the transparent deviant (*baker*) from 240 to 270 ms. This time window corresponds to the second half of the MMN response curve, which peaks at 165 ms. The significant effect in EEG covered the time window of 240–280 ms, corresponding to distinct spatial distributions for the two conditions: a negativity for the semantically transparent deviant (*baker*) in posterior electrodes and a positivity for the semantically opaque deviant (*beaker*) in central electrodes. No significant differences were found in the gradiometers.

At the source level, an effect between the two word deviants emerged in left anterior MTG, as seen in **Figure 6B**. From 260 to 270 ms, there was no main effect of condition (*F*_(1,14)_ = 2.05, *p* > 0.05), but a significant effect of ROI (*F*_(4,56)_ = 3.30, *p* < 0.05), and a significant condition by ROI interaction (*F*_(4,56)_ = 3.35, *p* < 0.05). Planned comparisons revealed increased activity for *beaker* compared to *baker* in left anterior MTG (*F*_(1,14)_ = 4.94,* p* < 0.05). In the right hemisphere, there was a significant effect of ROI (*F*_(4,56)_ = 2.70, *p* < 0.05), but there was no effect of condition (*F* < 1) or an interaction between the two factors (*F* < 1).

### INFLECTIONAL WORD CLASS CONTRAST: BAKES vs. BEAKS

In contrast to the [-er] forms, both word deviants with [-s] endings were morphologically complex and semantically transparent. At the mismatch response, peaking at 135 ms, the only difference between the [-s] deviants was linked to lateralization as described above (see **Figure 3B**).

### MONOMORPHEMIC STIMULI WITH EMBEDDED STEMS: BACON vs. BEACON

In contrast with multiple results obtained for affixed conditions, no significant differences in the MMN response were found at the sensor level between the non-affixed monomorphemic deviant stimuli.

## DISCUSSION

The aim of this study was to investigate the spatiotemporal pattern of morphological processing in the context of spoken word recognition, focusing on how neural activity within the bilateral frontal–temporal language network is modulated by the presence of a derivational or inflectional suffix. Results revealed language-specific responses that rapidly and automatically dissociated between words based on the presence of possible morphological complexity. All three conditions contained an embedded stem, and the addition of an ending that signaled either a potentially complex word or a non-affixed word resulted in distinct cortical distributions. For all conditions, the mismatch response peaked between 130 and 190 ms after the deviation point from the standard, and the source-level analysis revealed that neural activity within this time window showed a left-lateralized distribution in fronto-temporal regions. We focus on three major findings: the shift in the laterality of the brain response based on the grammatical properties of the deviants; the selectivity of the neural response for words compared to pseudowords, and the divergence between semantically transparent and opaque complex words.

### LATERALIZATION

The deviants all showed a left-lateralized distribution, but there was a significant shift in the degree of lateralization which was modulated by the presence of a potential affix. Both the [-s] and [-er] conditions showed increased left-lateralization within frontal and temporal regions compared to the [-on] condition during the mismatch response, and the lateralization for the affixed deviants was significantly greater than zero. This would suggest that the addition of a derivational or inflectional affix triggered increased engagement of left hemisphere fronto-temporal language regions, and this process occurred automatically once the suffix was present in the speech signal. This is in line with previous fMRI findings showing increased involvement of left-hemisphere perisylvian regions in morphological processing (Tyler et al., 2005; Lehtonen et al., 2006; Bozic et al., 2010), and supports the claim that the left-lateralized subsystem of the fronto-temporal network is specialized for processing of morphological complexity (e.g., Marslen-Wilson and Tyler, 2007). Importantly, unlike previous behavioral and fMRI results that could not speak to the timing of these events and were obtained using active tasks, the present study demonstrates that these fronto-temporal systems are triggered rapidly and automatically in the course of spoken word comprehension.

This increase in left hemisphere engagement was present for both suffix types, derivational and inflectional. Previous MMN research has not focused on derivationally complex forms; however source estimation from other studies examining morphological complexity and grammatical processing have demonstrated the key role of the left perisylvian areas in early stages of spoken word recognition (Shtyrov and Pulvermüller, 2002; Shtyrov et al., 2003). Furthermore, we found increased left-lateralization for both semantically transparent and opaque forms (*baker *and *beaker*), suggesting that morphological processing is triggered for any form containing morphological structure, regardless of word meaning. This is consistent with evidence from the visual domain showing automatic segmentation of word forms containing a stem and an affix, both behaviourally (Longtin et al., 2003; Rastle et al., 2004), and with MEG/EEG (Lavric et al., 2007; Morris et al., 2008; Lehtonen et al., 2011; Lewis et al., 2011), as well as fMRI evidence from spoken word comprehension demonstrating automatic decomposition of a stem and suffix (Tyler et al., 2002). Our findings are also in line with a dual-route account, in which parallel access through the full form as well as the constituents is engaged from early stages of recognition (Schreuder and Baayen, 1997). Word forms containing a stem and suffix would be initially decomposed; at a later stage the acceptability of the parsed form would be assessed, and semantically opaque forms would not be consistent with the decompositional route. However, the current study cannot speak directly to falsifying or strongly supporting dual-route accounts. Our results support initial morphological processing for all forms containing a potential suffix, which does not discount representation as whole forms.

We found additional laterality effects based on differences related to word class. The inflected word deviants contained a verb (*bakes*) and a noun (*beaks*). As both forms are semantically transparent and should be segmented into a stem and a suffix, they should not result in any differential processing due to the presence of morphological complexity. There were sustained laterality differences during the mismatch response, showing increased left-lateralization for the verb compared to the noun in frontal regions. The laterality analysis at the source level was in line with the evidence that verbs engage greater left perisylvian activity when they are inflected, which may be linked to the greater number of roles verbal affixes play in specifying number, tense and person (Tyler et al., 2004; Longe et al., 2007).

### LEXICALITY

The mismatch response showed a sensitivity to lexicality, with an increased response to words compared to the pseudoword which was strongest for the derived forms, i.e., [-er] deviants. The effect for the [-er] deviants appeared in left temporal sensors when comparing words vs. pseudowords, and at the source level was localized to left posterior STG. This is consistent with previous MMN findings showing a lexical enhancement effect (e.g., Pulvermüller et al., 2001), and indicates that lexical processing takes place automatically and does not require focused attention on the linguistic input. The presence of robust lexicality effects within left posterior temporal cortex during the mismatch response suggests that this area is involved in signaling acoustic changes (when deviants are sufficiently different from the standard) that are language-specific and in activating long-term cortical memory traces for stored words. In fMRI, left middle and superior temporal regions have been shown to play a key role in accessing stored lexical representations (Indefrey and Levelt, 2004; Hickok and Poeppel, 2007; Turken and Dronkers, 2011). Left STG was previously identified as underlying lexical MMN enhancement both in MEG (Shtyrov et al., 2005) and fMRI (Shtyrov et al., 2008).

The monomorphemic [-on] deviants also showed a left-lateralized distribution in temporal sensors, but the difference between word and pseudoword deviants appeared in the right hemisphere, showing increased activity for words. This suggests that both hemispheres respond to spoken words, although there may be a stronger left hemisphere involvement in this response. Whereas previously reported MMN lexicality effects were focused on the left temporal cortex, the potential role of right hemisphere generators has not been ruled out; furthermore, in at least one previous study a bilateral MMN response to concrete imageable nouns was linked to semantic stimulus features that are encoded by memory circuits encompassing both hemispheres (Pulvermüller et al., 2004). This is in line with extensive evidence for the involvement of the right hemisphere in language comprehension (e.g., Federmeier et al., 2008), as well as for increased bilateral engagement for morphologically simple words (Bozic et al., 2010). As we see in the laterality analysis, the monomorphemic [-on] deviants, which do not contain a potential suffix, show more bilateral fronto-temporal activity compared to the bimorphemic [-s] and [-er] forms, with the [-s] forms showing almost no right hemisphere activity at the peak of the MMN response (see **Figures 2B, C**). The combination of lexicality and laterality results point to the engagement of both the left and right temporal regions in lexical processing.

There was no significant lexicality effect in the inflected [-s] deviants, suggesting that the inflectional suffix was processed similarly for all forms, regardless of the meaning of the stem. This points to a specificity in the processing of the inflectional affix, which plays a grammatical role but does not alter the meaning of the stem (unlike derivational affixes, which change meaning and grammatical category). The inflectionally complex forms *bakes *and *beaks* do not require access to a separate representation from the stem, based on the argument that inflected forms are represented decompositionally (e.g., Pinker and Ullman, 2002). Thus, the same process of morphological segmentation should apply to both the words and pseudowords, suggesting that the [-s] suffix is triggering morphological processing as opposed to additional lexical processing.

### SEMANTIC TRANSPARENCY

The [-er] word forms varying in semantic transparency (*baker, beaker*) showed differential processing starting at 240 ms following the deviation point. We found increased processing of the semantically opaque word (*beaker*) which occurred more anteriorly, engaging left middle temporal cortex. We did not find similar amplitude differences between [-s] and [-on] pairs. This supports claims from the visual domain for a processing stage following blind segmentation which is constrained by word meaning, whereby the appropriateness of the segmentation is analyzed (Dominguez et al., 2004; Lavric et al., 2012). Semantically opaque forms such as *beaker *would require re-analysis since a decompositional meaning is not appropriate. The involvement of left anterior MTG points to additional processing demands required in accessing the appropriate meaning after an incorrect segmentation. Left MTG has been shown to be a key region in language comprehension (Turken and Dronkers, 2011), and anterior MTG in particular has been previously implicated in lexical retrieval (Damasio et al., 1996; Martin and Chao, 2001).

### AUTOMATICITY

In the present study, we extend the issue of automatic morphological processing to investigate how suffixed and non-suffixed forms are processed when attention is not focused on the stimuli and participants are not engaged in a stimulus-related task. Our results suggest that morphological segmentation is triggered automatically by the presence of a suffix, regardless of word meaning, activating a left-lateralized network of frontal and temporal regions. This would point to a primarily feedforward stimulus-driven process, driven by acoustic cues to morphological structure (*-s* or -*er* suffix). We report further evidence for automatic lexical processing, a finding which has been previously demonstrated when attention is not directed towards word identity (Price et al., 1996; Hinojosa et al., 2004). This does not disregard the crucial role of top-down processing, a relevant issue for understanding interactions between feedforward and feedback processes during word recognition – for instance, in examining task-relevant effects and how neural responses linked to morphological processing may be tuned by task demands (e.g., Wright et al., 2011). MEG and EEG could be beneficial in future studies in tracking neural activity across time between regions in the language network in order to investigate recurrent interactions between bottom-up and top-down processes during morphological and lexical processing.

Whilst using a limited set of stimuli, the MMN methodology offers a number of unique advantages because it (1) provides a tightly controlled method for studying neural processing of spoken words that are well-matched for acoustic and phonological similarity, (2) allows for examining language processes that occur independently of focused attention, and (3) allows for precise time-locking of brain activation to word recognition points in the spoken stimuli (Shtyrov and Pulvermüller, 2007). Variability in uniqueness point across words presents a challenge for examining large, controlled sets of stimuli in a typical event-related design. This is particularly important for suffixed words, since it makes it possible to control the point at which information about the stem and suffix is present in the speech signal across conditions. Importantly, at least in lexical and syntactic domains, initial MMN findings on rapid automatic processing could be confirmed in multi-item non-oddball designs (Hasting and Kotz, 2008; MacGregor and Shtyrov, 2013) when similarly rigorous stimulus matching was applied. In this way, focused MMN results could pave the way for further studies using more ecologically valid design. Future studies are needed to confirm the current MMN findings using other paradigms, including for example multi-item stimulus sequences with uniqueness point time-locking (cf. Leminen et al., 2011).

It is therefore crucial to consider how we can extrapolate to other words, and whether we can make conclusions about derivational, inflectional, and non-affixed words more generally from this study. The effects within this paradigm were robust and showed spatiotemporal patterns consistent with previous findings using morphologically simple and complex word forms. In order to further establish these results, additional studies looking at morphological complexity need to be performed using the MMN and other paradigms in the spoken domain. Given the limited morphological complexity of English in comparison with other languages, future studies are needed that will allow us to confirm these results using different stimuli in different languages. Using combined MEG and EEG and focusing analyses at the source level, it is possible to dissociate morphological processing from later stages involved in integration of semantic and syntactic aspects of the word, providing a more complete picture of the neural processing streams that support recognition of morphologically simple and complex words.

## CONCLUSION

We recorded automatic brain responses to acoustically and psycholingistically controlled sets of morphologically complex words, monomorphemic items and pseudoword control stimuli using combined MEG–EEG. In this study, we found:

• Automatic activation of lexical and morphological neural processes in response to complexity in spoken words as early as 130 ms after affix onset;
• Enhanced left lateralization of cortical activity for morphologically complex forms, which indicates involvement of left fronto-temporal cortical circuits;
• Stronger degree of left-lateralized processing for verb than noun stimuli;
• Modulation of automatic brain response to complex forms by their semantic coherence (transparency/opacity).

This study provides evidence that the spatiotemporal pattern of speech processing is modulated by the morphological status of the word ending. These results demonstrate processing of lexical and morphological features in the absence of focused attention, pointing to the key role that morphology plays in language comprehension.

## Conflict of Interest Statement

The authors declare that the research was conducted in the absence of any commercial or financial relationships that could be construed as a potential conflict of interest.

## Footnotes

1 We use here the standard linguistic definition of the morpheme as the minimal meaning-bearing linguistic unit (e.g., Matthews, 1991), distinguishing between ‘content’ morphemes like the stem {dark}, and grammatical morphemes like the derivational affix {-ness}.

2 A third type of morphological complexity in English involves compounds words (e.g., blackboard), composed of multiple roots as opposed to a root and affix, which are not assessed in the present study (but see MacGregor and Shtyrov, 2013, for related evidence on compound processing).
